# Northern Ohio Dental Association—Resolulutions of Respect to Dr. Jonathan Taft

**Published:** 1904-09

**Authors:** 


### NORTHERN OHIO DENTAL ASSOCIATION—RESOLUTIONS OF RESPECT TO DR. JONATHAN TAFT.

Whereas, In the death of Dr. Jonathan Taft, which occurred at Ann Arbor, Michigan, October 15, 1903, the dental profession has lost a most valuable member; one who gave generously of his knowledge for the advancement of his ideals; a noble manhood, ideal operations, and faithful services; a man, unselfish, gifted, kind, and true, using his God-given talents for a noble purpose,—

serving man; and one whose fidelity and Christian character must ever be an example, to the coming generations, of an ideal dentist; be it

*Resolved,* That we, his followers and members of the Northern Ohio Dental Association, in annual convention assembled, at Cleveland, June 7 to 9, 1904, express and record our appreciation of his noble life and generous contributions to the dental profession, and urge upon every member to live the life this gifted man lived, in order to bring to pass his conception of an ideal dental profession.

C. R. Butler,
Corydon Palmer,
F. S. Whitsler,
*Committee.*

C. D. Peck,
*Recording Secretary.*

